# Gene Expression Response to Sea Lice in Atlantic Salmon Skin: RNA Sequencing Comparison Between Resistant and Susceptible Animals

**DOI:** 10.3389/fgene.2018.00287

**Published:** 2018-08-03

**Authors:** Diego Robledo, Alejandro P. Gutiérrez, Agustín Barría, José M. Yáñez, Ross D. Houston

**Affiliations:** ^1^The Roslin Institute and Royal (Dick) School of Veterinary Studies, The University of Edinburgh, Edinburgh, United Kingdom; ^2^Facultad de Ciencias Veterinarias y Pecuarias, Universidad de Chile, Santiago, Chile; ^3^Aquainnovo S.A., Puerto Montt, Chile

**Keywords:** *Caligus rogercresseyi*, *Salmo salar*, aquaculture, disease, parasite, RNA-Seq, host–parasite, differential expression

## Abstract

Sea lice are parasitic copepods that cause large economic losses to salmon aquaculture worldwide. Frequent chemotherapeutic treatments are typically required to control this parasite, and alternative measures such as breeding for improved host resistance are desirable. Insight into the host–parasite interaction and mechanisms of host resistance can lead to improvements in selective breeding, and potentially novel treatment targets. In this study, RNA sequencing was used to study the skin transcriptome of Atlantic salmon (*Salmo salar*) parasitized with sea lice (*Caligus rogercresseyi*). The overall aims were to compare the transcriptomic profile of skin at louse attachment sites and “healthy” skin, and to assess differences in gene expression response between animals with varying levels of resistance to the parasite. Atlantic salmon pre-smolts were challenged with *C. rogercresseyi*, growth and lice count measurements were taken for each fish. 21 animals were selected and RNA-Seq was performed on skin from a louse attachment site, and skin distal to attachment sites for each animal. These animals were classified into family-balanced groups according to the traits of resistance (high vs. low lice count), and growth during infestation. Overall comparison of skin from louse attachment sites vs. healthy skin showed that 4,355 genes were differentially expressed, indicating local up-regulation of several immune pathways and activation of tissue repair mechanisms. Comparison between resistant and susceptible animals highlighted expression differences in several immune response and pattern recognition genes, and also myogenic and iron availability factors. Components of the pathways involved in differential response to sea lice may be targets for studies aimed at improved or novel treatment strategies, or to prioritize candidate functional polymorphisms to enhance genomic selection for host resistance in commercial salmon breeding programs.

## Introduction

Aquaculture is currently the fastest growing food industry (Food and Agriculture Organization of the United Nations, 2016) and is essential to meet increasing global demands for fish. However, the sustainability and prolonged success of any farming industry depends on effective disease prevention and control, and this tends to be particularly challenging for aquaculture. The aquatic environment and high stock density can expedite pathogen spread, which has historically resulted in periodic mass mortality events (Lafferty et al., 2015; Food and Agriculture Organization of the United Nations, 2017) and ongoing challenges in disease prevention and control. While biosecurity measures, vaccination, nutrition, and medicines all play vital roles for several diseases, selective breeding to produce more resistant and tolerant aquaculture stocks is rapidly becoming a key component of the battle to prevent these outbreaks (Yáñez et al., 2014a; Palaiokostas et al., 2016).

Sea lice, ectoparasites of the family Caligidae, are one of the major disease problems that the aquaculture industry is facing, and specifically for salmon farming. Atlantic salmon (*Salmo salar*) is the most important species in aquaculture with a production value of 14.7 billion US dollars in 2014 (Food and Agriculture Organization of the United Nations, 2016), therefore control of sea lice is a primary goal for the industry. Sea lice-related economic losses to worldwide salmonid aquaculture were estimated at ∼430 million USD per annum (Costello, 2009). Two lice species present the primary concerns for salmon farming: primarily *Lepeophtheirus salmonis* in the Northern Hemisphere and *Caligus rogercresseyi* in the Southern Hemisphere (Johnson et al., 2004). These copepods parasitize salmon during the marine phase of the lifecycle by attaching to their skin or fins, and feeding on the blood and tissue. This leads to open wounds which can facilitate the entry of other pathogens. The impaired growth and secondary infections cause significant negative animal welfare and economic impact (Frazer et al., 2012). Despite extensive use of both chemical (i.e., hydrogen peroxide, emamectin benzoate, organophosphates, pyrethroids, or benzoyl ureas) and non-chemical treatments (i.e., fresh water bath) to control sea lice, their negative impact on salmon aquaculture has increased in the past years (Torrisen et al., 2013), and various sea lice populations have been reported to be resistant to the most common chemicals available for therapeutic control, such as emamectin benzoate, azamethiphos (organophosphate), deltamethrin (pyrethroid), and even hydrogen peroxide (Aaen et al., 2015). Therefore, alternative methods to control sea lice are currently being studied, including the use of probiotics to reduce salmon attractiveness for sea lice (Jodaa et al., 2016) or cohabitation with lice-eating species (Imsland et al., 2014; Leclercq et al., 2014).

Knowledge of the interaction between salmon and sea lice can help devise more effective prevention and treatment strategies. Therefore, a lot of effort has been put in characterizing the host response to sea lice infestation (reviewed in Fast, 2014). Interestingly, the outcome of infestation varies for different salmonid species (Johnson and Albright, 1992), with coho salmon (*Oncorhynchus kisutch*) showing rapid inflammatory response and epithelial hyperplasia, leading to parasite encapsulation and more than 90% reduction in lice loads (Fast, 2014). In comparison, Atlantic salmon (*S. salar*) is highly susceptible to sea lice infestation and seemingly cannot mount a fully effective immune response (Fast, 2014). Comparative transcriptomics has shown that iron sequestration, increased expression of pattern recognition receptors such as c-type lectins and up-regulation of pro-inflammatory cytokines such as interleukin-1β are observed in salmon species resistant to sea lice (Sutherland et al., 2014). Interleukin-1β has also been implicated in successful responses to sea lice in other salmonid species such as pink salmon (*Oncorhynchus gorbuscha*) and coho salmon (Braden et al., 2012, 2015; Sutherland et al., 2015), and recently immunostimulant feeds up-regulating interleukin-1β in skin and spleen have shown some promising results to boost Atlantic salmon resistance to sea lice (Sutherland et al., 2017). While these studies have mainly focused on *L. salmonis*, similar findings have been observed in *C. rogercresseyi* infestation. For instance, comparative analyses of Atlantic and coho salmon parasitized with *C. rogercresseyi* showed that despite both showing up-regulation of pro-inflammatory genes, the response was highly specific, characterized in coho by an activation of the TH1 response (Valenzuela-Muñoz et al., 2016). Another study linked iron sequestration and depletion mechanisms to the Atlantic salmon immune response to *C. rogercresseyi* (Valenzuela-Muñoz et al., 2017).

A promising and potentially complementary approach to existing control measures is to exploit natural genetic variation in farmed salmon populations to breed stocks with enhanced resistance to the parasite. The presence of significant genetic variation for resistance to *C. rogercresseyi*, with heritability values ranging between 0.1 and 0.34, demonstrates the feasibility of improving this trait by selective breeding in Atlantic salmon (Lhorente et al., 2014; Yáñez et al., 2014b). Current evidence indicates that host resistance to sea lice in Atlantic salmon has a highly polygenic genetic basis, with little evidence for major QTL (Ødegård et al., 2014; Gharbi et al., 2015; Correa et al., 2016, 2017; Tsai et al., 2016). Therefore, genomic selection using genome-wide markers to predict lice resistant breeding values has been widely applied in commercial Atlantic salmon breeding programs, with a relative advantage compared to pedigree selection of 10–27% (Tsai et al., 2016; Correa et al., 2017). Understanding the underlying functional basis of genetic resistance to sea lice can lead to improved methods of selective breeding. For example, incorporating functional variants into genomic prediction models could help improve prediction accuracy, in particular for cross-population prediction (MacLeod et al., 2016). Functional annotation of reference genomes is pertinent to this process, and the emerging Functional Annotation of All Salmonid Genomes (FAASG) project (Macqueen et al., 2017) is aiming to improve genome annotation for Atlantic salmon (among other species). Further, the discovery of putatively causative genes and variants could, in the near future, lead to their introduction into populations or species where it has never been present through the use of genome editing, for example using CRISPR-Cas9 technology, which has been successfully applied in salmon to knockout two genes related to pigmentation (tyrosinase and solute carrier family 45 member 2) and the dnd (dead end) gene, producing albino (Edvardsen et al., 2014), and germ cell-free salmon (Wargelius et al., 2016), respectively.

Expression differences between Atlantic salmon resistant and susceptible families in response to *L. salmonis* for 32 immune genes suggested that resistant fish are better at avoiding immunosuppression (Holm et al., 2015). The same study found suggestive evidence that physical tissue barrier such as enhanced mucus production does not contribute to resistance (Holm et al., 2015). However, to our knowledge, the functional basis of genomic resistance to sea lice in Atlantic salmon has not been studied on a genome-wide scale, nor it has been explored in response to *C. rogercresseyi*. RNA sequencing can provide a first layer toward a holistic view of the host response to parasite infection, which in turn can highlight specific genes, pathways, and networks involved in the host–parasite interaction. RNA sequencing can also be used to identify single nucleotide polymorphisms in transcribed regions, and to assess the putative impact of those genetic markers on transcript and protein function. The effect of these markers on gene expression (and ultimately host resistance) can be assessed by allelic-specific expression or expression QTL studies, leading to a shortlist of candidate functional variants.

The overall aims of the current study were to compare the transcriptome profile of salmon skin at louse attachment sites and “healthy” skin (from the same fish), and to evaluate differences in these profiles between animals with varying levels of resistance to the parasite. To achieve this, challenged animals were classified into family-balanced groups according to resistance (based on high vs. low lice count) and growth during infestation, and RNA sequencing was performed on individual samples. By comparing resistant vs. susceptible samples, genes and pathways related to local immune response and host resistance were identified, and their potential role discussed.

## Materials and Methods

### Experimental Design

2,668 Atlantic salmon (*S. salar*) pre-smolts (average weight: 136 g) from 104 families from the breeding population of AquaInnovo (Salmones Chaicas, Xth Region, Chile), were experimentally challenged with *C. rogercresseyi* (chalimus II–III). This population will be used for a future study on sea lice resistance genetic architecture and genomic selection. Briefly, infestation with the parasite was carried out by using 13–24 copepodids per fish and stopping the water flow for 6 h after infestation. Eight days after the infestation fish were euthanized and fins from each fish were collected and fixed for processing and lice counting. 42 samples from 21 fish from 6 different families (2–5 fish per family) were selected for RNA sequencing (Supplementary File S1) based on the traits of interest (number of sea lice attached to their fins and growth during challenge). Skin samples (both from attachment sites and health skin) were obtained from each animal and stored in RNAlater at 4°C for 24 h, and then at -20°C until RNA extraction for sequencing.

### RNA Extraction and Sequencing

For all the 42 samples a standard TRI Reagent RNA extraction protocol was followed. Briefly, approximately 50 mg of skin was homogenized in 1 ml of TRI Reagent (Sigma, St. Louis, MO, United States) by shaking using 1.4 mm silica beads, then 100 μl of 1-bromo-3-chloropropane (BCP) was added for phase separation. This was followed by precipitation with 500 μl of isopropanol and posterior washes with 65–75% ethanol. The RNA was then resuspended in RNAse-free water and treated with Turbo DNAse (Ambion). Samples were then cleaned up using Qiagen RNeasy Mini kit columns and their integrity was checked on Agilent 2200 Bioanalyzer (Agilent Technologies, United States). Thereafter, the Illumina Truseq mRNA stranded RNA-Seq Library Prep Kit protocol was followed directly. Libraries were checked for quality and quantified using the Bioanalyzer 2100 (Agilent), before being sequenced on three lanes of the Illumina Hiseq 4000 instrument using 75 base paired-end sequencing at Edinburgh Genomics, United Kingdom. Raw reads have been deposited in NCBI’s Sequence Read Archive (SRA) under Accession No. SRP100978.

### Read Mapping

The quality of the sequencing output was assessed using FastQC v.0.11.5.^1^ Quality filtering and removal of residual adaptor sequences was conducted on read pairs using Trimmomatic v.0.32 (Bolger et al., 2004). Specifically, Illumina specific adaptors were clipped from the reads, leading, and trailing bases with a Phred score less than 20 were removed and the read trimmed if the sliding window average Phred score over four bases was less than 20. Only reads where both pairs were longer than 36 bp post-filtering were retained. Filtered reads were mapped to the most recent Atlantic salmon genome assembly (ICSASG_v2; GenBank Accession No. GCF_000233375.1; Lien et al., 2016) using STAR v.2.5.2b (Dobin et al., 2013), the maximum number of mismatches for each read pair was set to 10% of trimmed read length, and minimum and maximum intron lengths were set to 20 bases and 1 Mb, respectively. Uniquely mapped paired-reads were counted and assigned to genes (NCBI *S. salar* Annotation Release 100) using FeatureCounts (Liao et al., 2014), included in the SourceForge Subread package v.1.5.0. Only reads with both ends mapped to the same gene were considered in downstream analyses.

### Differential Expression

Differential expression analyses and gene functional and pathway enrichment analyses were performed using R v.3.3.1 (R Core Team, 2014). Gene count data were used to estimate differential gene expression using the Bioconductor package DESeq2 v.3.4 (Love et al., 2014). Briefly, size factors were calculated for each sample using the median of ratios method and count data was normalized to account for differences in library depth, next gene-wise dispersion estimates were fitted to the mean intensity using a parametric model and shrinked toward the expected dispersion values, finally a gegative binomial model was fitted for each gene and the significance of the coefficients was assessed using the Wald test. The Benjamini–Hochberg false discovery rate (FDR) multiple test correction was applied, and transcripts with FDR < 0.05 and absolute log_2_ fold change values (FC) > 0.5 were considered differentially expressed genes. Hierarchical clustering and principal component analyses were performed to visually identify outlier samples that did not cluster close to other samples in the same category (lice attachment site or healthy skin), which were then removed from the analyses as sampling errors could not be discounted. PCA plots were created using the R package factoextra.^2^

### Pathway Enrichment

Gene Ontology (GO) enrichment analyses were performed using Blast2GO v.4.1 (Conesa et al., 2005). Briefly, genes showing >10 reads in >90% of the samples were annotated against the manually curated protein database Swiss-Prot (Bairoch et al., 2004) and GO terms were assigned to them using Blast2GO. GO enrichment for specific genes lists was tested against the whole set of expressed genes using Fisher’s exact test. GO terms with ≥5 DE genes assigned and showing a Benjamini–Hochberg FDR corrected *p*-value < 0.05 were considered enriched. Kyoto Encyclopedia of Genes and Genomes (KEGG) enrichment analyses were performed using KOBAS v3.0.3 (Wu et al., 2006). Briefly, genes showing >10 reads in >90% of the samples were annotated against KEGG protein database (Kanehisa and Goto, 2000) to determine KEGG Orthology. KEGG enrichment for specific gene lists was tested by comparison to the whole set of expressed genes using Fisher’s exact test. KEGG pathways with ≥5 differentially expressed (DE) genes assigned and showing a Benjamini–Hochberg FDR corrected *p*-value < 0.05 were considered enriched.

## Results and Discussion

### Disease Challenge

A total of 2,632 fish belonging to 105 families from a commercial breeding program were challenged with *C. rogercresseyi* copepods, and euthanized for sampling 8 days post-challenge. Average lice burden per fish was 38 ± 16, and the estimated heritability of sea lice load was 0.28 ± 0.04 (unpublished results), therefore the differences in sea lice counts between fish has a genetic component. Fish were selected for RNA sequencing based on the traits of resistance, measured as number and concentration of lice per fish, and weight and length gain since the start of the challenge, which may reflect the ability of the fish to cope with the infestation. The selected fish allowed for 8 vs. 8 comparisons between family-matched fish showing differential resistance (26.2 ± 5.5 vs. 54.9 ± 13.5 sea lice per fish) and differential growth during infestation (7.0 ± 4.3 vs. 28.8 ± 12.3 weight gain percentage). A total of 42 samples (21 fish, skin from sites of louse attachment and healthy skin) were sequenced, resulting in an average of ∼27.9 ± 2.7 million reads per sample. After trimming, these were aligned against the salmon reference genome (ICSASG_v2; GenBank Accession No. GCF_000233375.1; Lien et al., 2016) and levels of gene expression were estimated according to the official salmon genome annotation (NCBI *S. salar* Annotation Release 100). An average of 19 M trimmed reads per sample were assigned to genes and used for downstream analyses of gene expression. All raw sequence data is available in NCBI’s SRA under BioProject Accession No. SRP100978, and may be a useful contribution to the functional annotation of all salmonid genomes initiative (FAASG; Macqueen et al., 2017).

### Louse Attachment Sites Versus Healthy Skin

**FIGURE 1:**
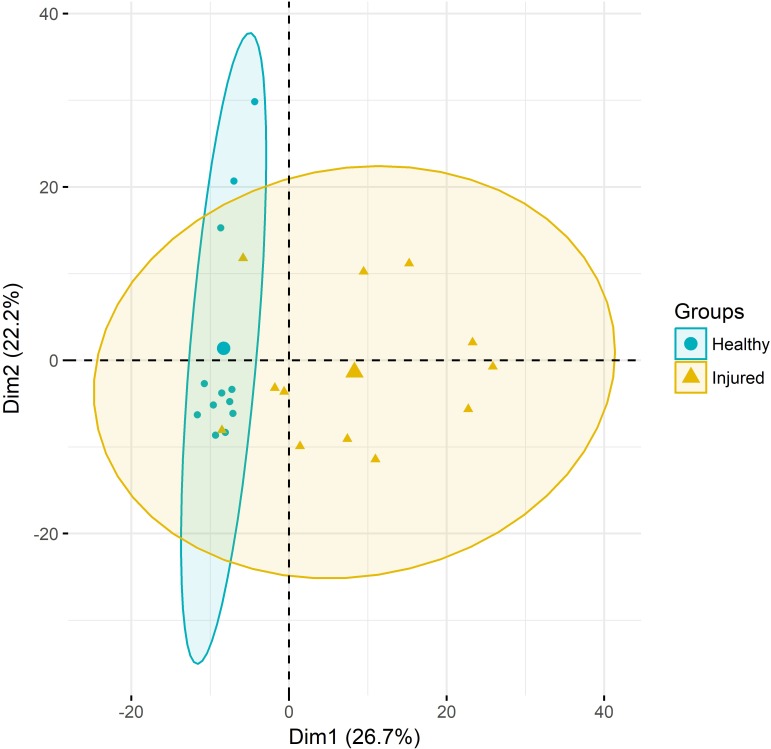
Principal component analyses. RNA-Seq samples clustered according to their gene expression. Ellipses represent 95% confidence intervals.

**FIGURE 2:**
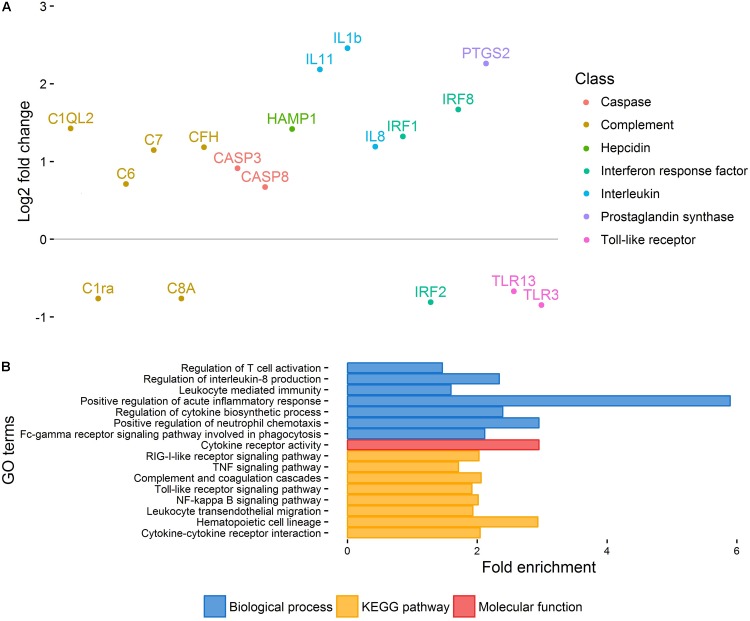
Healthy vs. injured skin. **(A)** Important immune-related genes showing differential expression between healthy and injured skin. Genes have been arbitrarily positioned along the *x*-axis. **(B)** Selection of GO terms enriched amongst DE genes between healthy and injured skin.

Principal component analysis of gene expression (**Figure 1**) revealed a relatively clear cluster of healthy skin samples, while lice-attachment samples were more scattered, probably reflecting variation in the individual response to sea lice. Differential expression between healthy and louse attachment sites resulted in 4,355 DE genes (Supplementary File S2), with a higher number of up-regulated (more expressed in attachment sites) than down-regulated genes (*n* = 3,114 vs. *n* = 1,241). Among these DE genes were well-known components of the innate immune response such as interleukins, interferon response factors and complement components (**Figure 2A**). GO term and KEGG pathway analyses (Supplementary File S2) revealed a clear enrichment of immune pathways and functions among the up-regulated genes (**Figure 2B**), highlighting a localized immune response strongly related to cytokine activity. A similar scenario has been observed in other salmonids such as coho salmon where resistance to sea lice has been associated with early inflammation in skin and head kidney, which results in epithelial hyperplasia and often parasite encapsulation and removal of the sea lice within 2 weeks (Johnson and Albright, 1992; Fast et al., 2002). In pink salmon, an early and high expression of pro-inflammatory genes (IL-8, TNFα-1, and IL-1β) has been suggested as a mechanism of rapid louse rejection (Fast et al., 2007). The classical complement pathway has also been linked to resistance of host fish to parasitic copepod infection (Fast, 2014). The results presented here indicate that despite a marked up-regulation of the local inflammatory response and complement pathway in Atlantic salmon, in part resembling the response of coho salmon or pink salmon, this does not seem to be sufficient to successfully respond to the louse attachment and feeding.

In addition to the expected innate immune response observed above, cell division related processes were also clearly up-regulated at louse attachment sites, and well-characterized genes involved in tissue repair such as fibroblast growth factor-binding protein 1 and Epigen showed significant differences between lice attachment sites and healthy skin (FC > 3). Several genes related to the cell matrix and cell adhesion also had higher expression at attachment sites (i.e., cadherin-13, integrin alpha-2, desmoplakin, or various keratin and collagen genes). Cell proliferation is the main response to skin wounds in fish (Iger and Abraham, 1990), and these results are consistent with those previously found in the early response to *L. salmonis* (Skugor et al., 2008). Several mucins were also found to have higher expression at attachment sites, pointing toward increased mucus production and secretion, which can also be a typical response to wounding in fish (Fast, 2014).

### Resistance

Resistance, measured as number of sea lice per fish, was evaluated using two different approaches: correlation between gene expression and sea lice loads, and differential expression between family-matched fish showing high and low sea lice loads.

#### Correlation Between Gene Expression and Sea Lice Loads

We studied the correlation of gene expression and sea lice counts in healthy skin and sea lice attachment sites. Genes showing *r* > |0.75| with sea lice counts were considered of interest (Supplementary File S3).

The expression levels of five immune receptors in healthy skin were positively correlated with sea lice loads (**Table 1**). Macrophage mannose receptor 1 (MRC1) shows the highest positive correlation with number of sea lice (*r* = 0.87), and also the highest expression difference between louse attachment vs. healthy skin (FC = 4.79). MRC1 is a c-type lectin receptor, expressed in macrophages, dendritic cells, and skin in humans. MRC1 plays a role both in innate and adaptive immunity and also acts as a recognition receptor for different pathogens such as bacteria, virus, or fungi (East and Isacke, 2002). C-type lectin receptor A (*r* = 0.81; FC = -1.34) is another lectin receptor involved in antigen recognition and immune response (Geiktenbeek and Gringhuis, 2009). Lectins such as MRC1 and CLEC4E have been found to be induced by glucosinolate-enriched feeds in Atlantic salmon, which also reduced lice counts between 17 and 25% (Holm et al., 2016), and are also up-regulated in response to sea lice in the more resistant pink salmon species (Sutherland et al., 2014). Lectins have been reported to activate the immune system in response to parasites in several different species (Vázquez-Mendoza et al., 2013; Hoving et al., 2014), therefore modulation of these genes represents a possible route to enhance Atlantic immune responses to sea lice. Two immune receptors were negatively correlated with number of sea lice, CD97 (*r* = -0.84) and suppressor of cytokine signaling 5 (SOCS5; *r* = -0.76). CD97 regulates cytokine production and T-cell activation and proliferation (Capasso et al., 2006; Abbott et al., 2007); while SOCS5 is part of the cytokine-mediated signaling pathway, and acts as a negative regulator of inflammatory response and other immune-related pathways (Seki et al., 2002). Since, it was not possible to take skin samples prior to infection, it is difficult to distinguish between cause and effect; i.e., it is plausible that the negative correlation of these genes with number of sea lice is simply indicating that the immune system of the host responds proportionally to the degree of lice infestation. Nonetheless, the data support a major role for these genes in the host response to sea lice, and the differences in lice count between fish has a genetic component, to which these genes may contribute.

**Table 1:** Immune receptors showing correlation with sea lice counts.

Gene	Full name	Corr. (*r*)	GO terms
MRC1	Macrophage mannose receptor 1	0.87	Cellular response to interferon gamma Cellular response to interleukin-4
CLEC4E	C-type lectin receptor A	0.81	Innate immune response Positive regulation of cytokine secretion
CR2	Complement receptor type 2	0.78	Innate immune response Complement activation, classical pathway
LIFR	Leukemia inhibitory factor receptor	0.78	Cytokine-mediated signaling pathway
CD28	T-cell-specific surface glycoprotein CD28	0.77	Positive regulation of inflammatory response to antigenic stimulus Cytokine biosynthethic process
SOCS5	Suppressor of cytokine signaling 5	-0.76	Cytoine-mediated signaling pathway Negative regulation of inflammatory response
CD97	CD97 antigen	-0.84	Inflammatory response

Amongst genes without a (well-known) immune function, there was an association between SUMO1 (*r* = 0.76) and SUMO3 (*r* = -0.91) expression and sea lice loads. Small ubiquitin-like modifier (SUMO) proteins are small proteins similar to ubiquitins that are covalently attached to other proteins to modify their function. According to the gene expression data, SUMO1 seems to be preferred over SUMO3 in salmon upon sea lice infestation. Although post-translational modifications have been barely explored in fish, in mice SUMOylation has been shown to be involved in modulation of host innate immune response to pathogens (Decque et al., 2016). SUMOylation is also a very active field of research in plants, where SUMO is known to be involved in many important processes such as plant response to environmental stresses, including pathogens (Park et al., 2011). It would be interesting to further study the role of SUMO in modulating Atlantic salmon responses to sea lice.

The results were markedly different in lice-attachment sites (Supplementary File S3), and congruent with differential expression between lice-attachment and healthy skin, with inflammatory genes such as toll-like receptor 12 or caspase 3 showing high correlations with sea lice loads. Similarly, one of the sox9 paralogs (sox9a) was also highly correlated with lice loads. Sox9 has a pro-proliferation function in human epidermal keratinocytes (Shi et al., 2013), and therefore this transcription factor is probably promoting wound healing in sea lice attachment sites. Finally, the gene hepcidin-1 is also correlated with sea lice counts in lice-attachment sites. Hepcidin is a regulator of iron metabolism, which as mentioned in the introduction has been associated with response to *C. rogercresseyi* (Valenzuela-Muñoz et al., 2017).

#### Differential Expression Between High and Low Sea Lice Loads

**FIGURE 3:**
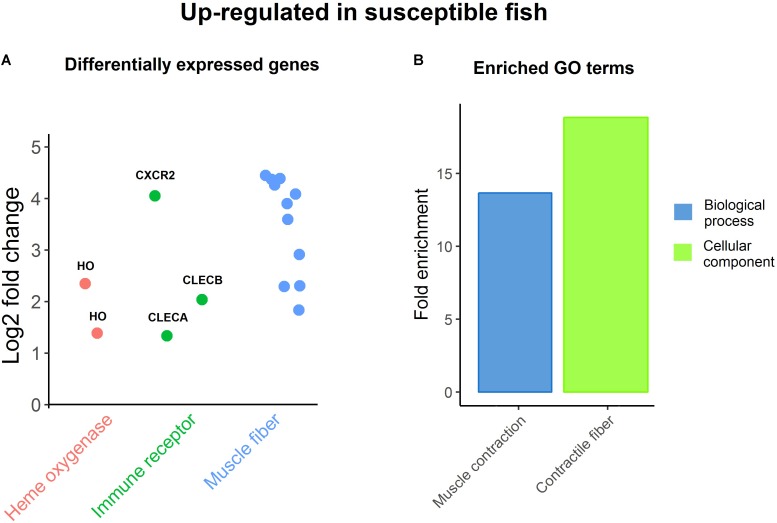
Up-regulation in susceptible fish. **(A)** Genes DE between resistant and susceptible fish, being up-regulated in the latter. **(B)** Enriched GO terms amongst DE genes up-regulated in susceptible fish.

The samples for RNA sequencing were chosen to enable 8 vs. 8 comparison between family-matched fish (three families with two fish per group, two families with one fish) with high and low values for resistance (26.2 ± 5.5 vs. 54.9 ± 13.5 sea lice per fish). There were 43 genes significantly differentially expressed between resistant and susceptible fish (Supplementary File S4). All but one were from comparison of healthy skin samples between the two groups, which seems to suggest that the differences in resistance are systemic rather than local to louse attachment sites. The susceptible group had higher expression levels for genes involved in muscle contraction like troponins and myosins, which was also highlighted by GO enrichment analyses (**Figure 3**). Myosins and troponins have previously been identified as genes that respond to sea lice attachment in salmon skin (Holm et al., 2015). Further, *Caligus* infection is known to induce increased enzyme activity in muscle tissue (Vargas-Chacoff et al., 2017), and behavioral changes in the fish such as flashing and jumping are associated with ectoparasite removal (Furevik et al., 1993; Magnhagen et al., 2008). It has been recently reported that inactivity or reduced swimming activity contribute to resistance to sea lice (Bui, 2017), so it is possible that the high lice counts of susceptible fish in this study are due to higher activity levels with associated expression of muscle contraction related genes. In turn, high lice burden can provoke behavioral responses increasing fish activity, which results in the up-regulation of muscle genes, increasing the expression differences between resistant-passive-low lice fish and susceptible-active-high lice fish.

Two heme oxygenase genes, encoding enzymes, which catalyze the degradation of heme, also had higher expression levels in susceptible samples (**Figure 3**), which is consistent with the positive correlation with lice loads of the iron-sequestration gene hepcidin. These genes have been previously shown to be up-regulated in response to *Caligus* infection (Valenzuela-Muñoz and Gallardo-Escárate, 2017). Importantly, iron availability was found to be reduced in the highly resistant species pink salmon infected with *L. Salmonis* (Sutherland et al., 2014), and hematocrit and anemia were also found to be reduced in chum salmon (*Oncorhynchus keta*) in response to sea lice (Jones et al., 2007). It is therefore plausible that the more effective reduction of iron availability in Atlantic salmon (perhaps behaving more similarly to the resistant pink salmon) might be related to increased resistance to sea lice.

Finally, three immune receptors showed higher expression in susceptible samples (**Figure 3**); C-X-C chemokine receptor type 2 is a receptor for IL-8, its binding causes activation of neutrophils; while C type lectin receptors A (also found to be positively correlated with sea lice counts in healthy skin) and B are leptin receptors with an important role in pathogen recognition and immunity (Geiktenbeek and Gringhuis, 2009), as previously discussed. While it is clear that resistance and host response to sea lice is multifactorial in nature, these genes related to muscle contraction, iron availability and immunity may be targets for functional validation in future studies, and for cross-referencing with genome-wide association analyses to identify candidate causative genes and variants.

### Growth During Infestation

Differences in weight gain percentage from the start to the end of the trial were also investigated. Weight gain during infestation did not show any significant correlation with initial weight (*r* = -0.27, *p* = 0.10), sea lice counts (*r* = 0.12, *p* = 0.45) or sea lice density (*r* = 0.19, *p* = 0.24) in our dataset, and the means for these three traits are not significantly different between our groups showing differential growth during infestation (*t*-test *p*-values > 0.35). Family-matched fish (8 vs. 8; three families with two fish per group, two families with one fish) with differential weight gain during infestation (7.0 ± 4.3 vs. 28.8 ± 12.3 weight gain percentage) were compared. A total of 24 and 1 genes were found differentially expressed between fish showing high and low weight gains in healthy and sea louse attachment site samples, respectively (Supplementary File S5). The gene differentially expressed in injured skin, solute carrier family 15 member 1 (SLC15A1), also showed the lowest *p*-value and highest FC in healthy skin (FC = 3.38, *p* = 0.003). The SLC15A1 protein is a membrane transporter that mediates the uptake of dipeptides and tripeptides, in humans this gene is expressed in the intestinal epithelium and plays a major role in protein absorption (Adibi, 1997). Another interesting DE gene is myogenic regulatory factor 6 (MYF6; FC = 0.72, *p* = 0.04). Myogenic regulatory factors are transcription factors that regulate muscle development (Perry and Rudnick, 2000); in *Senegalese sole* decreased expression of these factors was observed in fast muscle when fed with a high-lipid content diet, which caused reduced growth (Campos et al., 2010). While skin is unlikely to be a highly suitable tissue to study genes underlying fish growth during sea lice infestation, both myogenic factors and increased nutrient absorption, and specifically MYF6 and SLC15A1, are good candidates to better understand growth impairment differences under sea lice infestation.

## Conclusion

The results of this study highlight that the early gene expression response of Atlantic salmon to sea lice involves up-regulation of many different components of the immune system (inflammatory response, cytokine production, TNF and NF-kappa B signaling and complement activation) along with tissue repair activation. The comparison of resistant vs. susceptible animals highlighted enrichment of pathways related to fish activity, iron availability and receptors modulating pathogen recognition and immune response. Overall, this study contributes to an improved understanding of Atlantic salmon early response to sea lice in skin, and into the gene expression profiles underpinning genetic resistance to sea lice in salmon. The identified pathways and genes may be targets for future studies aimed at development of new treatments, vaccines, or prevention strategies. The data can also be cross-referenced with high power genome-wide association studies to help prioritize putative causative genes and variants that have potential to improve genomic selection programs for genetic improvement of resistance to this industry’s most serious disease.

## Data Availability

The raw reads generated for this study have been deposited in NCBI’s Sequence Read Archive (SRA) under Accession No. SRP100978.

## Ethics Statement

The lice challenge experiments were performed under local and national regulatory systems and were approved by the Animal Bioethics Committee (ABC) of the Faculty of Veterinary and Animal Sciences of the University of Chile (Santiago, Chile), Certificate No. 01-2016, which based its decision on the Council for International Organizations of Medical Sciences (CIOMS) standards, in accordance with the Chilean standard NCh-324-2011.

## Author Contributions

RH, JY, and DR were responsible for the concept and design of this work and drafted the manuscript. AB managed the collection of the samples. AG performed the molecular biology experiments. DR performed bioinformatic and statistical analyses. All authors read and approved the final manuscript.

## Conflict of Interest Statement

JY was supported by Aquainnovo S.A. The remaining authors declare that the research was conducted in the absence of any commercial or financial relationships that could be construed as a potential conflict of interest.

## Footnotes

1 http://www.bioinformatics.babraham.ac.uk/projects/fastqc/

2 http://www.sthda.com/english/rpkgs/factoextra/
